# Assessment of Residual Bone Level Around Explanted Implants and Description of the Systemic and Local Characteristics. A Retrospective Study

**DOI:** 10.1111/clr.70004

**Published:** 2025-07-16

**Authors:** Rodrigo Martin‐Cabezas, Norbert Cionca, Catherine Giannopoulou

**Affiliations:** ^1^ Division of Regenerative Dental Medicine and Periodontology University Clinics of Dental Medicine Geneva Switzerland

**Keywords:** explantation, marginal bone loss, peri‐implantitis, treatment outcome

## Abstract

**Objectives:**

The study aimed to evaluate the residual bone level of explanted implants and to report local and systemic characteristics observed in patients who underwent implant explantation.

**Material and Methods:**

A total of 494 administrative records were initially retrieved from the database. After elimination of duplicates, 464 medical records of patients who underwent implant explantation between 2005 and 2021 in thirteen private clinics in Switzerland were screened. Information regarding implant history, local (oral health, periodontal and prosthetic status) and systemic (systemic diseases, medication, and smoking) characteristics was assessed through the medical records and the radiographic data. ISRCTN registry (ISRCTN10631004).

**Results:**

In total, 399 patients and 521 implants were included in the analysis. Peri‐implantitis was the most frequent reason for explantation (62%). Explantation was mainly performed in cases of advanced peri‐implantitis with a mean bone loss of 62.92% of the implant length. Most patients had radiographic bone loss around the remaining dentition with a bone loss/age ratio of 0.74, presented compromised oral health, and 47.80% of them were smokers. Moreover, only 56.12% of patients reported being in good systemic health.

**Conclusion:**

Implant explantations were typically performed once bone loss reached approximately 60% of the implant length. They were most often observed in advanced cases of peri‐implantitis and in patients with high‐risk profiles for periodontal breakdown, such as a history of periodontal disease or heavy smoking habits.

## 1 Introduction

Peri‐implantitis treatment has shown high survival rates of the affected implants (Roccuzzo et al. 2018); however, the outcomes of these interventions are less predictable than those for periodontal treatments, with recurrence rates reaching 45% after 5 years (Carcuac et al. 2020). In advanced cases, bone loss can lead to implant failure (Monje and Nart 2022). Consequently, one of the most significant prognostic factors in peri‐implant treatment is the level of initial bone loss (de Waal et al. 2016). When bone loss exceeds 50% of the length of the implant, explantation has been recommended (Monje and Nart 2022; Sinjab et al. 2018), as treatment beyond this threshold becomes 20 times less predictable compared to implants with bone loss < 25% (Ravida et al. 2022).

Determining the residual bone level beyond which an implant cannot be preserved and explantation is recommended, remains challenging (Martin‐Cabezas and Giannopoulou 2024). Recent studies suggest a threshold of two‐thirds of the implant length (66.2%) as the point at which practitioners are more likely to proceed with extraction. However, limited scientific data exist on this topic, and larger cohorts are needed to analyze the influence of additional factors (Wentorp et al. 2021).

Some risk factors for peri‐implantitis are well‐established, such as history of periodontal disease, poor plaque control, or lack of follow‐up, whereas others, such as diabetes and smoking, until recently remained inconclusive (Schwarz et al. 2018). The periodontal condition of adjacent teeth can influence the microbiological colonization of the implants (Eick et al. 2016), emphasizing the importance of controlling periodontal pockets in patients with implant‐supported restorations. Additionally, periodontal bone loss relative to age is a critical factor for understanding the progression rate of periodontitis (Tonetti et al. 2018). This factor may also elucidate the history of periodontitis, which has been associated with peri‐implantitis (Carra et al. 2022), particularly in severe cases (Roccuzzo et al. 2014) and in noncompliant patients (Roccuzzo et al. 2023). Moreover, prosthetic restorations can hinder access to plaque control (Serino and Strom 2009), and specific prosthetic factors, such as emergence angles, emergence profiles, or splinting, have been associated with increased peri‐implantitis prevalence (Yi et al. 2020). Only recently, there is growing evidence of the influence of systemic diseases, such as hyperglycemia and smoking, on the prevalence of peri‐implant diseases (Monje et al. 2023).

Given this context, the aim of this retrospective study is to analyze the residual bone level of implants at the time of explantation and to describe the reasons for this procedure. We further assessed the local (dental, periodontal, prosthetic and implant‐related factors) and systemic (age, gender, systemic diseases, medications, and smoking) characteristics of patients who underwent implant explantation. By defining a patient profile for implant loss, this study aims to assist practitioners in strengthening monitoring and preventive treatment.

## 2 Material and Methods

### 2.1 Study Design and Setting

The data used in this retrospective study were extracted from patient records, identified from the billing database of a group of 13 private dental clinics in Switzerland between January 2005 and December 2021. The study was conducted in accordance with the Declaration of Helsinki on human studies, following approval from the Ethics Committee for Research of Geneva (Switzerland) under protocol reference number 2022‐00151 for the use of health‐related data without individual consent, in accordance with Swiss Law Art. 34 LRH, Art. 37‐40 ORH. The study protocol was registered at the ISRCTN registry (ISRCTN10631004) and the results were reported according to the STROBE guidelines (Vandenbroucke et al. 2007).

### 2.2 Participants

To be eligible for the study, the medical record had to include: (i) data related to adult patients with an explanted dental implant during the inclusion period; (ii) radiographic data at explantation; and (iii) the reason for explantation.

The exclusion criteria were: (i) unclear reason for explantation; (ii) incomplete radiographs (radiographs that do not allow measurement of the entire implant or are unusable due to exposure/distortion, etc.); (iii) implants placed for orthodontic reasons, needle‐shaped implant, temporary implants; (iv) non‐identifiable position of the explanted implant, or (v) document confirming patient's refusal.

### 2.3 Collected Data

The medical records were screened, and the following data were collected when available:

- At the patient level: age at the time of explantation, gender, history of periodontal treatment, systemic conditions, medication use, and tobacco consumption.
- At the implant level: date of explantation, position of the implant, implant lifespan, type of implant, reason for explantation, initial periodontal diagnosis, periodontal probing depth at explantation, and frequency of follow‐up prior to the explantation.

### 2.4 Radiographic Assessment

**FIGURE 1:**
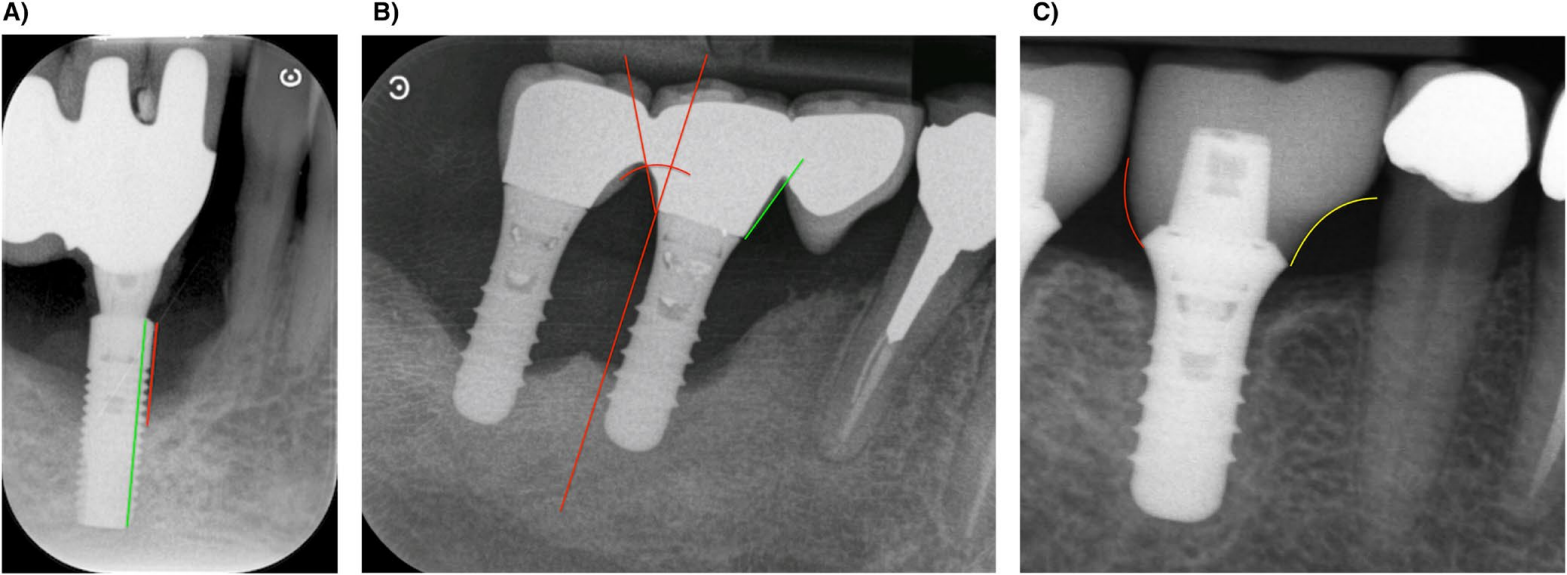
Assessment of radiographic data. (A) The bone loss was assessed in millimeters from the shoulder of the implant to the apical part of the bone defect (red line). The relative bone loss was reported as a percentage of the total implant length (green line) affected by the defect. (B) Emergence angle of the prosthetic reconstruction measured as the angle between the tangent of the transitional contour relative to the long axis of the implant (red lines). (B and C) Emergence profile of the prosthetic restauration was classified as droit (green line), convex (red line), or concave (yellow line).

Periapical, panoramic, and CBCT X‐ray files were analyzed with the CS imaging software version 7.0.3 and 8 (Carestream Dental) by the same examiner (R.M.C.) who assessed the following:

- At the patient level: number of missing teeth, number of implants, the decayed, missing, and filled teeth (DMFT) index, bone loss at teeth, and ratio bone loss/age.
- At the implant level: marginal bone loss at explantation (mm) from the implant shoulder to the most apical part of the bone defect, relative bone loss at explantation (%) assessed as percentage of the implant length with peri‐implant bone loss (Figure 1), type of the peri‐implant bone defect as follows: class I (infraosseous), class II (supracrestal/horizontal) or class III (combined) and severity of the defect as follows: Grade S (Slight: 3–4 mm/< 25% of the implant length), Grade M (Moderate: 4–5 mm/≥ 25%–50%) or Grade A (Advanced: > 6 mm/> 50%) (Monje et al. 2019). Additionally we recorded the type, extent and splinting of the prosthesis, number of abutments, position of the implant on the prosthetic restoration, presence and position of a cantilever, presence of micro‐gap between the implant and the prosthesis, emergence angle and profile at mesial and distal as defined by the Glossary of Prosthodontic Terms, 9th edition (“The Glossary of Prosthodontic Terms: Ninth Edition,” 2017), crown‐implant ratio and type of antagonist and proximity to neighboring anatomical structures (tooth/implant, sinus, nasal cavity, inferior alveolar nerve).

Intra‐rater reliability was not formally assessed in this study. However, all radiographic evaluations were performed by a single experienced examiner following a standardized protocol to minimize variability.

### 2.5 Variables and Outcomes

To achieve the study's objectives, we focused on several clinical and radiographic variables. The primary objectives were to evaluate: (i) the amount of residual bone level around implants at the time of explantation (Outcome 1) and the prevalence of peri‐implantitis as the main reason for explantation (Outcome 2).

Furthermore, we describe several local characteristics (implant‐related factors, periodontal and dental factors, prosthetic reconstruction factors) and systemic characteristics related to the patient (systemic conditions, medications, tobacco consumption) (Outcome 3 and 4, respectively).

### 2.6 Statistical Analysis

The entire cohort was analyzed for explantation reasons, and further sub‐analysis was performed only on the patients who underwent an explantation due to peri‐implantitis. Quantitative variables were summarized using descriptive statistics, including means, standard deviations (SD), averages, and ranges, depending on the variable distribution. Categorical data were expressed as frequencies and percentages. When some data was missing, the patient or the implant concerned was not included in that specific analysis. The sample size for each analysis is, in consequence, reported on each table.

## 3 Results

### 3.1 Participants

**FIGURE 2:**
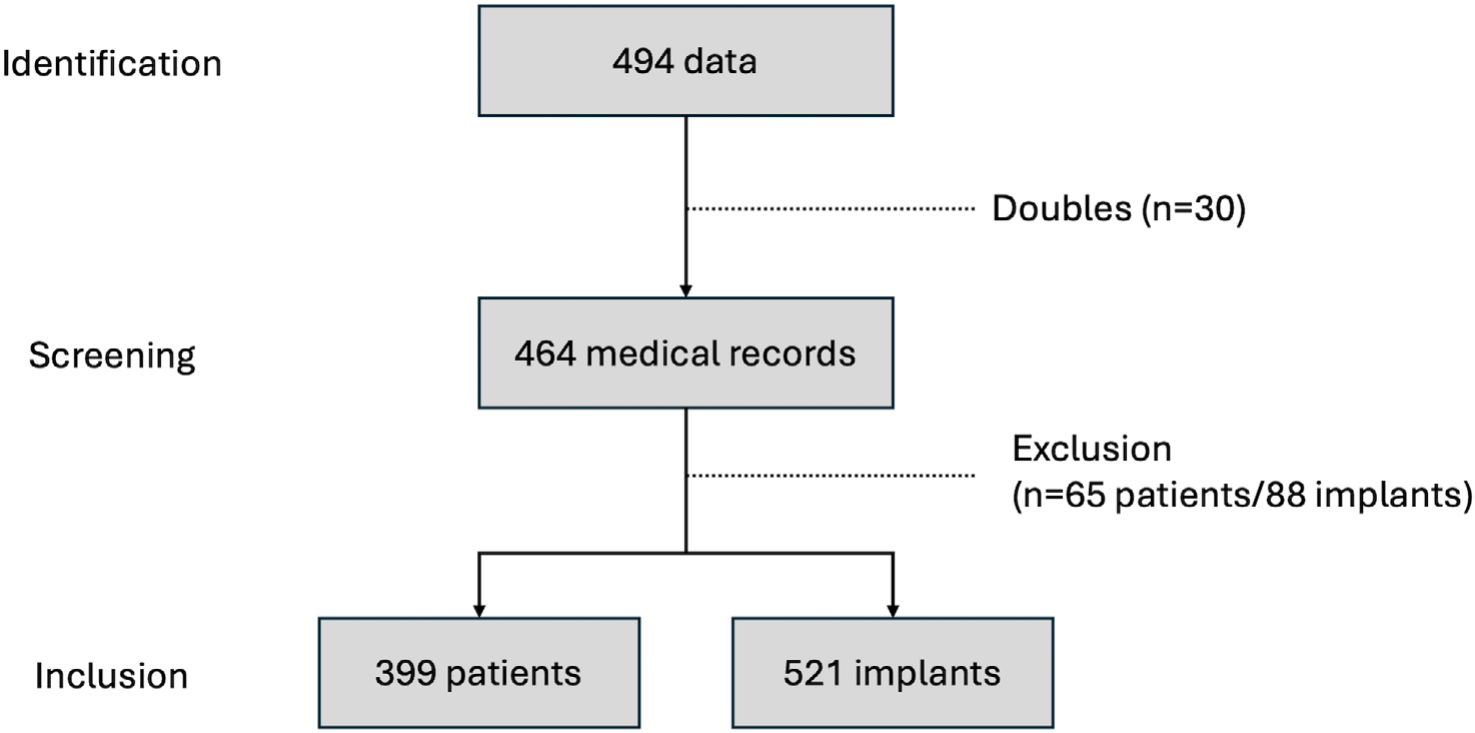
Flow chart of study inclusion.

A total of 494 patient files were identified as having undergone implant explantations through the billing database. After resolving duplicates (*n* = 30), which included cases where the same patient was treated in multiple clinics, a total of 464 medical files corresponding to 609 implants were screened. Following exclusions, 399 patients and 521 implants were included in the analysis. Figure 2 presents the study inclusion flowchart, and Table 1 details the reasons for exclusion.

**TABLE 1:** Reasons for exclusion.

	Patients	Implants
Orthodontic/needle‐shaped implant or apex	31 (47.69%)	34 (36.56%)
Absence of X‐rays	17 (26.15%)	24 (25.81%)
Reason for explantation no identifiable	5 (7.69%)	9 (9.68%)
X‐rays no exploitable	2 (3.08%)	7 (7.53%)
Position of the implant no identifiable	4 (6.15%)	4 (4.30%)
Other reasons^a^	6 (9.23%)	10 (11.36%)

^a^ Billing code error (*n* = 2), implant fell out on its own (*n* = 2), explantation requested by the patient despite the absence of any clinical justification (*n* = 6).

### 3.2 Descriptive Data

**FIGURE 3:**
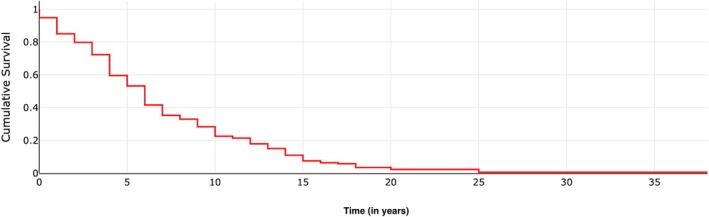
Time to implant explantation: Survival analysis.

Demographic characteristics of the included population (all reasons of explantation included) and the subset with peri‐implantitis‐related explantations are presented in Table 2. The distribution of explantations was equal between both sexes. The age at the time of explantation ranged from 17 to 88 years, with a mean age of 60.40 years. Only one patient was younger than 20 years old, and in this case, the implant was extracted due to primary failure. Some implants were removed within the first year after placement, whereas others remained functional for more than 35 years, with a mean implant lifespan of 5.14 years. As shown in Figure 3 the survival rate dropped sharply within the first 6 years, followed by a more gradual decrease. Explanted implants were more frequently located in the maxilla than in the mandible (58.51% and 41.49%, respectively) and posterior sextants were more commonly affected than anterior sites.

**TABLE 2:** Demographic data of the included population.

	All explantations *n* (%/SD)	Explantations due to peri‐implantitis *n* (%/SD)
Gender	*N* = 399 patients	*N* = 242 patients
Male	193 (48.37%)	112 (46.28%)
Female	206 (51.63%)	130 (53.72%)
Number of explantations per patient	*N* = 399 patients	*N* = 242 patients
Explantations per patient	1.31	1.33
Patients with 1 implant explanted	323 (80.95%)	191 (78.93%)
Patients with 2 implants explanted	52 (13.03%)	34 (14.05%)
Patients with 3 implants explanted	12 (3.01%)	8 (3.30%)
Patients with 4 implants explanted	8 (2.01%)	7 (2.89%)
Patients with 5 implants explanted	1 (0.25%)	0 (0%)
Patients with more than 5 implants explanted	3 (0.75%)	2 (0.83%)
Patient age at explantation (per implant)^a^	*N* = 521 implants	*N* = 323 implants
Years	60.40 (13.17)	60.87 (13.66)
Position of the implant	*N* = 521 implants	*N* = 323 implants
Maxillary	317 (60.84%)	189 (58.51%)
Mandibular	204 (39.15%)	134 (41.49%)
Sextant 1	105 (20.15%)	65 (20.12%)
Sextant 2	99 (19%)	58 (17.96%)
Sextant 3	113 (21.69%)	66 (20.43%)
Sextant 4	87 (16.7%)	58 (17.96%)
Sextant 5	41 (7.87%)	31 (9.60%)
Sextant 6	76 (14.59%)	45 (13.93%)
Implant lifespan	*N* = 337 implants	*N* = 173 implants
Years	5.14 (5.75)	7.15 (5.84)

^a^ For patients with multiple explantations, age was recorded individually for each implant at the time of explantation.

When the number of explantations per patient was analyzed, the mean value was 1.31. Although most patients (80.95%) underwent only one explantation, some had multiple implants removed: two explantations (13.03%); 3 (3.01%); 4 (2%) or more (1%). A few individuals experienced a cluster of explantations, with more than five implants removed.

### 3.3 Main Results

#### 3.3.1 Outcome 1. Bone Level at Explantation Time

The residual bone level at the time of explantation was analyzed only for the peri‐implantitis patients, and after exclusion of implants presenting 100% bone loss, provided more accurate data regarding the explantation decision. The type and severity of the defect were also classified according to Monje et al. (2019) (Monje et al. 2019). The relative bone loss ranged from 18.84% to 94.21% with a mean value of 62.92%, corresponding to 6.76 mm of bone loss. Most explantations (50.79%) involved combined supra‐ and infra‐bony defects, and 80.42% of the peri‐implantitis cases were classified with advanced severity (Table 3). The number of residual bone walls could not be assessed as only radiographs were available in the medical records.

**TABLE 3:** Bone loss at the explantation time in the peri‐implantitis group after exclusion of implants with bone loss = 100%.

	*n* (%)	SD	Max	Min
Bone loss at explantation (*N* = 192)				
Bone loss (mm)	6.76	2.09	12.2	1.9
Relative bone loss (%)	62.92	16.49	94.21	18.84
Type of defect (*N* = 192)^a^				
Class I	44 (23.28%)			
Class II	49 (25.93%)			
Class III	96 (50.79%)			
Severity of the defect (*N* = 192)^a^				
Slight	1 (0.53%)			
Moderate	36 (19.05%)			
Advanced	152 (80.42%)			

^a^ As described by Monje et al. (2019).

**FIGURE 4:**
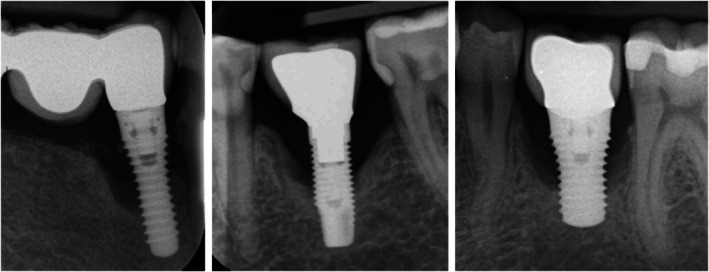
Examples of explanted implants included in the study with approximately 60% of peri‐implant bone loss.

Figure 4 shows three cases in which explantation was due to peri‐implantitis (showing approximately 60% bone loss).

#### 3.3.2 Outcome 2. Prevalence of Peri‐Implantitis Among the Reasons for Explantation

The explanations were classified based on their primary reason (Table 4). Peri‐implantitis was the most reported reason, accounting for 62% of cases. This was followed by primary failure (16.51%), aseptic loosening (11.32%) and fractures (5.95%).

**TABLE 4:** Distribution according to the reason for explantation (*N* = 399 patients/521 implants).

	Patients	Implants
Peri‐implantitis	242	323 (62%)
Primary failure	75	86 (16.51%)
Aseptic loosening	58	59 (11.32%)
Fracture	27	31 (5.95%)
Other reasons^a^	21	22 (4.22%)

*Note:* Some patients had multiple implants removed for different reasons.

^a^ Other reasons include malposition (*n* = 16), vestibular bone dehiscence (*n* = 2), aesthetics (*n* = 2), lack of function (*n* = 1), and pain (*n* = 1).

#### 3.3.3 Outcome 3. Local Characteristics: Implant, Prosthetic, and Dental/Periodontal Status

Different local factors were analyzed in the peri‐implantitis group when data were available (Table 5). Implant‐related factors revealed a mean diameter of 4.21 mm, with a low proportion of narrow implants (11.88%). Additionally, 30.21% of the implants had a length of less than 10 mm. Limited information was available regarding other factors, such as the manufacturer of the implant, implant surface, or surgical techniques, especially for patients referred externally. This limitation prevented a detailed analysis of these factors, as they might have been influenced by the local clinical practices.

**TABLE 5:** Local characteristics of the explanted implants in the peri‐implantitis group (calculated per implant).

	*N* (%)
Implant related	
Mean diameter (*N* = 101)	4.21 mm
Diameter < 4.0 mm	12 implants (11.88%)
Mean length (*N* = 96)	9.75 mm
Length < 10 mm	29 implants (30.21%)
Type of prothesis (*N* = 323)	
Fixed	231 (71.52%)
Removable	61 (18.89%)
Temporary	8 (2.48%)
No prosthesis	23 (7.12%)
Type of antagonist (*N* = 277)	
Natural teeth	156 (48.30%)
Fixed implant‐supported prosthesis	39 (12.07%)
Removable prosthesis	33 (10.22%)
Natural teeth + fixed implant‐supported prosthesis	29 (8.98%)
Natural teeth + removable prosthesis	13 (4.02%)
No antagonist	7 (2.17%)

At the time of explantation, some patients had lost all their natural teeth, whereas for others, the implant replaced only a single missing tooth (Table 6). The mean number of missing teeth in this population was 14.41, with an average of 4.74 implants per patient. The mean DMFT index was 22.75, and this value remained high when the 25 completely edentulous patients were excluded (mean DMFT index = 22.05). Radiographic analysis revealed that nearly every patient had clinical attachment loss, with bone loss averaging 45% of the root length. Furthermore, the bone loss/age ratio was 0.74, representing a moderate rate of periodontal progression (Tonetti et al. 2018). Despite this, only 18% of patients reported a history of periodontal treatment.

**TABLE 6:** Periodontal and dental situation in the peri‐implantitis group at the time of explantation (calculated per implant).

	*N* (%)	SD	Max	Min
Dental conditions at explantation				
Number of missing teeth (*N* = 228)	14.41	8.24	28	1
Number of implants (*N* = 232)	4.74	3.55	16	1
DMFT index (*N* = 211)	22.75	5.80	28	3
Periodontal conditions at explantation				
Mean PPD (*N* = 76)	3.84 mm	1.12	6.68	2.08
% PPD > 3 mm (*N* = 76)	36.93%	21.83	78.07	2
% PPD > 5 mm (*N* = 76)	17.33%	17.42	59.65	0
Bone loss at teeth (*N* = 275)	254 (92.36%)			
% Root with bone loss at teeth (*N* = 222)	45.66%	26.01	100	0
Bone loss/age (*N* = 222)	0.74	0.46	2.33	0
History of periodontal treatment (*N* = 218)	58 (17.96%)			

When initial periodontal charting was available (*N* = 52), periodontitis was classified by stages (Papapanou et al. 2018): stage I (0%), stage II (2%), stage III (58%), and stage IV (40%), and by grades: grade A (0%), grade B (50%), and grade C (50%). However, the presence of deep pockets (> 5 mm) at the time of explantation was limited to 17.33% of the patients.

Prosthetic restoration data highlighted a predominance of fixed prostheses (71.52%) compared to removable ones (18.89%). Natural teeth were the most common antagonists (48.30%), followed by implant‐supported fixed prostheses (12.07%) and removable prostheses (10.22%) (Table 5). Analyzing the characteristics of fixed prostheses (Table 7), explantations were almost equally distributed between unitary (42.42%) and splinted restorations (57.58%). Poor adaptation between the prosthetic reconstruction and the implant was observed in 24% of radiographs, and 12% of implants had a combined restoration supported by both teeth and implants. Although single‐unit restorations were the most common (43%), bridges with at least four crowns were the second most frequent (23.48%). Explantations were more prevalent in implants with distal cantilever or straight emergence profiles.

**TABLE 7:** Fixed prosthesis in the peri‐implantitis group (calculated per implant) related to number of crowns, abutments, and emergence profile.

Fixed prosthesis	*n* (%)
Number of patients (*N* = 231 implants)	176 patients
Unitary	98 implants (42.42%)
Splinted	133 implants (57.58%)
Mesial	36 (27.07%)
Middle	27 (20.30%)
Distal	70 (52.63%)
Cantilever (*N* = 182 implants)	
No	162 (89.01%)
Mesial	9 (4.95%)
Distal	11 (6.04%)
Microgap (*N* = 225 implants)	56 (24.88%)
Number of abutments (*N* = 230 implants)	
Mean number of abutments	2.06
1 abutment	100 (43.47%)
2 abutments	74 (32.17%)
3 abutments	28 (12.17%)
≥ 4 abutments	28 (12.17%)
Type of abutment (*N* = 230 implants)	
Implants	202 (87.82%)
Implants + teeth	28 (12.17%)
Number of crowns (*N* = 230 implants)	
Mean number of crowns	2.78
1 crown	100 (43.47%)
2 crowns	37 (16.09%)
3 crowns	39 (16.96%)
≥ 4 crowns	54 (23.48%)
Mesial emergence angle (*N* = 215 implants)	30.41
30°	120 (55.81%)
≥ 30°	95 (44.19%)
Distal emergence angle (*N* = 215 implants)	30.53
30°	121 (56.27%)
≥ 30°	94 (43.72%)
Mesial emergence profile (*N* = 215 implants)	
Concave	59 (27.44%)
Straight	136 (63.25%)
Convex	20 (9.30%)
Distal emergence profile (*N* = 215 implants)	
Concave	70 (32.56%)
Straight	137 (63.72%)
Convex	8 (3.72%)
Crown:Implant ratio (*N* = 226 implants)	0.91

The characteristics of removable prostheses are detailed in Table 8. In two cases, the medical records did not provide sufficient information to determine whether the implant supported a complete or partial prosthesis. As a result, these two patients were excluded from the detailed analysis of prosthesis‐related characteristics. There were 59 explanted implants with removable prostheses. Forty‐nine were included in complete reconstructions and ten in partial prostheses. It was not possible to assess the number of abutments for four implants because the periapical X‐rays and the medical file did not allow for all the abutments to be visualized. The analysis concerning the number of abutments was therefore performed on 55 implants. Most of the removed implants served as abutments for complete removable prostheses (83.05%), predominantly in the maxilla (71.43%). One‐third of these implants were splinted. Regarding the number of abutments, four was the most frequent configuration (34.55%). Importantly, no explantations occurred in mandibular sites with fewer than two implants, whereas 19.35% of implants were removed in maxillary positions with fewer than four implants. Lastly, a small proportion of implants (7%) served as abutments for combined restorations involving both teeth and implant‐supported prostheses.

**TABLE 8:** Removable prosthesis in the peri‐implantitis group (calculated per implant).

Removable prosthesis	*N* (%)
Number of patients (*N* = 59 implants)^a^	44 patients^a^
Explanted implants included on partial prosthesis	10 implants (16.95%)
Explanted implants included on complete prosthesis	49 implants (83.05%)
Indication for complete prothesis	
Maxillary	35 (71.43%)
Mandibular	14 (28.57%)
Splinting	20 (33.90%)
Number of abutments (*N* = 55 implants)	
Mean number of abutments of the reconstruction	3.55
1 abutment	2 (3.66%)
2 abutments	15 (27.27%)
3 abutments	9 (16.36%)
4 abutments	19 (34.55%)
≥ 5 abutments	10 (18.18%)
Complete maxillary with < 4 implants (*N* = 31)	6 (19.35%)
Complete mandibular with < 2 implants (*N* = 14)	0 (0%)
Type of abutments (*N* = 57 implants)	
Implants	53 (92.98%)
Implants + teeth	4 (7.02%)

^a^ Two medical files did not allow us to determine if the implant was an abutment of a complete or partial prosthesis. Therefore, analysis was performed on 44 patients/59 implants instead of 46 patients/61 implants.

#### 3.3.4 Outcome 4. Systemic Characteristics: Systemic Conditions, Medication, and Tobacco Consumption

Systemic characteristics related to the patients' profile were analyzed in the peri‐implantitis group and are described in Table 9. Regarding systemic health, 56.12% of patients reported being in good health. Certain conditions prevalent in more than 10% of the population included cardiovascular diseases, hypertension, and high cholesterol (14.77%, 22.78%, and 10.97%, respectively). Diabetes was present in 7.17% of patients.

**TABLE 9:** Systemic characteristics in the peri‐implantitis group (calculated per patient).

	*n* (%/SD)
Systemic health (*N* = 237)	
Healthy	133 (56.12%)
Diabetes	17 (7.17%)
Cardiovascular	35 (14.77%)
Hypertension	54 (22.78%)
Cholesterol	26 (10.97%)
Medications (*N* = 234)	
Antidiabetic	16 (6.84%)
Antiplatelet	33 (14.10%)
Anticoagulant	13 (5.56%)
Antihypertensive	50 (21.37%)
Statin	24 (10.26%)
Antidepressant	16 (6.84%)
Smoking (*N* = 182)	
Yes	87 (47.80%)
Former smoker	15 (8.24%)
Cigarettes per day (*N* = 62)	13.89 (7.72)

Medication use corresponded to the reported health conditions. Antiplatelet agents, antihypertensive medications, and statins were used by more than 10% of patients, whereas antidiabetic medications were taken by 6.84% of patients.

Data on tobacco consumption (*N* = 182) revealed a high proportion of smokers (47.80%) with an average consumption of 13.89 cigarettes per day, ranging from 2 to 40. Additionally, 8.24% of the patients identified as former smokers; however, details regarding the duration since quitting and the quantity previously smoked were inconsistently reported.

### 3.4 Other Analyses

#### 3.4.1 Follow‐Up Visits

The proportion of patients with at least two follow‐up appointments per year for scaling or supporting periodontal therapy was approximately 25% in the year preceding the explantation. This percentage dropped significantly, with only 10% of patients receiving consistent follow‐up within the 5‐year period before the explantation. Notably, 37% of patients had no follow‐up visits during the year prior to the explantation.

#### 3.4.2 Relationship With Neighboring Anatomical Structures

The proximity of implants to neighboring anatomical structures was analyzed. Nearly 30% of implants were positioned within 1 mm of the maxillary sinus. Smaller proportions were found for other structures: neighboring teeth or implants (12.38%), the inferior alveolar nerve (6.19%) and the nasal fossa (2.78%).

## 4 Discussion

The present retrospective study aimed to identify the bone level at the time of explantation and to describe the profile of patients who underwent implant explantation. Implants were mainly explanted for peri‐implantitis reasons, with a mean bone loss of 62.92% of the implant length, indicating severe peri‐implantitis cases (Monje et al. 2019). These results agree with those from a recent study that reported a mean bone loss of 66.2% at explantation time (Wentorp et al. 2021) and with the current scientific evidence regarding the thresholds for implant explantation (Martin‐Cabezas and Giannopoulou 2024). Furthermore, clinical studies have shown a decrease in successful treatment when bone loss around implants increases (de Waal et al. 2016). Hence, implants with more than 50% of bone loss at baseline increase the risk of implant failure after peri‐implant surgical treatment by 20‐fold compared to implants where bone loss was < 25% of the length (Ravida et al. 2022). Moreover, in a recent retrospective analysis on the peri‐implantitis surgical prognostic factors, implants with bone loss ≥ 60% had a poorer survival prognosis when compared with implants with < 40% (Romandini et al. 2024). These studies have shown an additional effect when simultaneous risk factors are present, such as smoking (de Waal et al. 2016), modified implant surface, or suppuration at baseline (Romandini et al. 2024).

Peri‐implantitis was the main reason for explantation in this study, accounting for 62% of implants, followed by primary failure, aseptic loosening, and fractures. This aligns with the results of previous reports where explantations were performed in 64.5% (Gargallo‐Albiol et al. 2021) and 59.9% of patients (Roy et al. 2020).

The population of this study presented a highly compromised oral health with a mean of 14.41 missing teeth and a DMFT score of 22.75 (DMFT index of 22.05 after exclusion of fully edentulous patients). Peri‐implantitis has been associated with factors related to poor oral health factors such as the number of missing teeth, untreated interproximal caries or fillings adjacent to implants (Vilarrasa et al. 2021). Moreover, the population of our study had several implants per patient (4.74 ± 3.55). Peri‐implantitis was found more frequently in patients with more than five implants (Passoni et al. 2014) and similar results were reported for patients with two or more implants (Gurgel et al. 2017).

Radiographic bone loss around teeth was present in 92.36% of the patients. This highlights the association between the history of periodontitis and peri‐implantitis (Schwarz et al. 2018). Longitudinal studies have shown a high risk of peri‐implantitis in patients with a history of periodontitis (Karoussis et al. 2003), especially in patients with severe periodontitis (Roccuzzo et al. 2012). Similar results were confirmed when periodontal charting was present, with most patients diagnosed with stages III (58%) and IV (40%) and grades B (50%) and C (50%). Moreover, the mean bone loss/age ratio was 0.74, which implies a high risk of periodontitis progression (Tonetti et al. 2018). Recently, stage IV and bone loss/age ratio > 1 were associated with peri‐implantitis occurrence in a longitudinal study (OR = 41.29 and OR = 8.87, respectively) (Romandini et al. 2025). Only 18% of the patients reported a history of periodontal treatment; taking into consideration the link between both diseases, this context could increase the proportion of implants explanted for peri‐implantitis reasons.

Most frequently, explanted implants were restored by single‐unit crowns (43%), followed by bridges with at least four crowns (23.48%). Long extension prostheses such as full arch bridges have been frequently associated with peri‐implantitis (Romandini et al. 2025). In addition, maladaptation between the implant and the crown was radiographically evident at 24.88% of the explanted implants in our study. The gap can lead to a bacterial leakage, which would imply a peri‐implant inflammatory reaction (Candotto et al. 2019).

No differences in the distribution of angle or emergence profile were found compared to other studies which reported angles ≥ 30° (Yi et al. 2020) or ≥ 45° (Corbella et al. 2023) as a risk of peri‐implantitis. This fact could be due to the different types of implants in this study, including bone‐ and tissue‐level implants. In fact, when Yi et al. (2020) analyzed only tissue‐level implants, there was no significant association between the prosthetic factors and peri‐implantitis (Yi et al. 2020). In our study, the lack of information regarding the type of connection prevented further analyses.

Only limited information exists in the literature regarding the link between peri‐implantitis and systemic diseases with, however, contradictory results. For instance, a retrospective study found a significant impact on the early onset of peri‐implantitis in patients with diabetes mellitus, osteoporosis, and hypertension; however, another study failed to find any association (Astolfi et al. 2022). The most frequently reported systemic disease in our study was hypertension, affecting 22.78% of the population, followed by cardiovascular diseases (14.77%). Similar values for hypertension (28.6%) have already been reported in patients with peri‐implantitis (Anderson et al. 2020). Moreover, a recent meta‐analysis concluded that the presence of cardiovascular diseases increases the risk of peri‐implantitis (Chu et al. 2023).

Current evidence regarding the role of smoking as a risk factor for peri‐implantitis is inconclusive (Schwarz et al. 2018). When data regarding smoking habits was available, 87 patients out of 182 reported smoking (47.80%), mainly heavy smokers (> 10 cigarettes per day) (Ravida et al. 2020) with an average consumption of 13.89 cigarettes per day. Smoking has been associated with peri‐implantitis incidence (OR = 7.84) in a recent longitudinal prospective study (Romandini et al. 2025), and heavy smokers have been associated with radiographic bone loss (Carral et al. 2021).

Finally, irregular follow‐up or no follow‐up was frequent during the years before the explantation. It has been reported that patients who are compliant with supporting therapy are less likely to develop peri‐implantitis (Monje et al. 2017). In a study reassessing the peri‐implant health of mucositis patients after 5 years, the lack of follow‐up was associated with a 43.9% frequency of peri‐implantitis compared to 18% of cases when the patient received at least 1 follow‐up appointment per year (Costa et al. 2012).

The mean strength of our study is the sample size, which provides data with external validity. Moreover, a patient profile based on multiple local and systemic characteristics is described. In addition, including several private practices can shed light on the clinical practice trends, without focusing on a unique practitioner‐based philosophy of treatment.

However, there are also some limitations inherent in the retrospective design of our study that should be clarified to correctly interpret the results. This design does not allow for determining the causal relationship of any of the analyzed factors/characteristics. For example, if most implant patients are over the age of 60, older individuals will naturally be overrepresented among those experiencing implant explantation. However, this overrepresentation does not necessarily imply a greater risk of implant loss associated with older age. Furthermore, different radiographic methods were available in the medical files, and information on the presence of systemic diseases was self‐reported. Moreover, the morphology of the defect could not be assessed as only a few files included probing pocket depths or intra‐surgical images. Consequently, only the type of defect and the severity could be analyzed. Other factors such as plaque level, keratinized mucosa dimensions, and previous peri‐implant treatment were not consistently reported in the medical files, preventing their analysis.

Further studies should include control groups to determine the potential risk factors for implant loss. Moreover, studies should focus on the combined potential risk factors of peri‐implantitis to determine a high‐risk profile and to increase clinical efforts to maintain peri‐implant health, thus avoiding explantation as ultimate treatment.

## 5 Conclusion

Peri‐implantitis was the most frequent cause of implant explantation. Explantations were typically performed once bone loss reached approximately 60% of the implant length. They were most often observed in advanced cases of peri‐implantitis and in patients with high‐risk profiles for periodontal breakdown, such as those with a history of periodontal disease or heavy smoking habits.

## Author Contributions

**Rodrigo Martin‐Cabezas:** conceptualization, data curation, formal analysis, visualization, writing – original draft, methodology, investigation, writing – review and editing, validation, project administration. **Norbert Cionca:** writing – review and editing, validation. **Catherine Giannopoulou:** conceptualization, writing – review and editing, supervision, methodology, validation, project administration, formal analysis, visualization.

## Conflicts of Interest

The authors declare no conflicts of interest.

## Data Availability

The data that support the findings of this study are available on request from the corresponding author. The data are not publicly available due to privacy or ethical restrictions.
